# Comparative study on the analgesic effect of vortioxetine and other antidepressants in the streptozotocin mouse model of painful diabetic neuropathy

**DOI:** 10.1177/17448069251367596

**Published:** 2025-08-02

**Authors:** Serena Notartomaso, Roxana Paula Ginerete, Francesca Liberatore, Ferdinando Nicoletti, Valeria Bruno, Giuseppe Battaglia

**Affiliations:** 1 Department of Molecular Pathology, Neuropharmacology Unit, I.R.C.C.S. Neuromed, Pozzilli, Italy; 2 Department of Physiology and Pharmacology, Sapienza University, Rome, Italy

**Keywords:** Antidepressants, diabetic neuropathy, streptozotocin, vortioxetine

## Abstract

Preclinical studies show that vortioxetine displays a robust analgesic activity in models of neuropathic pain. Here, we compared the effect of a 2-week treatment with vortioxetine, duloxetine, amitriptyline, fluoxetine, and paroxetine (all injected i.p. at the daily dose of 10 mg/kg) on mechanical and thermal pain thresholds, risk-taking behaviour and depressive-like behaviour in the streptozotocin (STZ) mouse model of painful diabetic neuropathy. Vortioxetine, duloxetine and amitriptyline reduced mechanical pain in diabetic mice, with vortioxetine displaying the greatest efficacy. In contrast, paroxetine and fluoxetine were inactive. Vortioxetine, duloxetine, amitriptyline and paroxetine were also effective in enhancing thermal pain thresholds in diabetic mice. Induction of diabetes did not affect risk-taking behaviour in the light-dark box test but enhanced depressive-like behaviour in the tail suspension test. All antidepressants, with the exception of amitriptyline, reversed depressive-like behaviour, whereas paroxetine unexpectedly reduced risk-taking behaviour in diabetic mice. We conclude that vortioxetine may offer therapeutic value for alleviating pain in diabetic neuropathy, particularly in patients with comorbid depression.

## Introduction

Antidepressants that inhibit both serotonin and noradrenaline reuptake, for example, amitriptyline, duloxetine and venlafaxine, are first-line drugs in the treatment of neuropathic pain^1–4^ and they are widely used in the treatment of comorbid pain and depression.^5^ The high rate of drug resistance and the cardiovascular adverse effects of serotonin/noradrenaline reuptake inhibitors (SNRIs) and tricyclic antidepressants (TCAs)^6^ makes the use of these drugs in the treatment of neuropathic pain suboptimal, and encourages the search for novel therapeutic targets or the repositioning of drugs that are already marketed for the treatment of depression and are not indicated for neuropathic pain.

One of the reasons why selective serotonin reuptake inhibitors (SSRIs) show low efficacy in the treatment of neuropathic pain is that serotonin becomes hyperalgesic in chronic pain. Selective serotonin depletion in rostral ventromedial medulla (RVM) neurons projecting to the spinal cord induced analgesia in rats with neuropathic and inflammatory pain, without altering pain thresholds in healthy controls.^7^ The hyperalgesic action of serotonin in chronic pain may result from a hyperactive state of serotonergic neurons caused by the epigenetic silencing of inhibitory interneurons in the RVM raphe nuclei.^8^ The pain enhancing action of serotonin is largely mediated by the activation of 5-HT_3_ receptors in the spinal cord.^9–14^ Accordingly, pharmacological blockade of 5-HT_3_ receptors alleviates neuropathic pain in rodents and humans.^10,15^

Vortioxetine is a multimodal antidepressant, which displays high potency as a 5-HT_3_ receptor antagonist.^16^ In addition, vortioxetine inhibits the high affinity serotonin transporter and behaves as a full agonist of 5-HT_1A_ receptors, a partial agonist of 5-HT_1B_ receptors and an antagonist of 5-HT_1D_ and 5-HT_7_ receptors.^17,18^ Vortioxetine is a first-line drug in the treatment of depression,^19^ but has no clinical indication for the treatment of neuropathic pain. We found that a 3-week treatment with vortioxetine caused robust analgesia in the chronic constriction injury (CCI) model of neuropathic pain in mice.^20^ This original observation was followed by an increasing number of preclinical studies showing the analgesic activity of vortioxetine in preclinical models of chemotherapy-induced neuropathy,^21^ painful diabetic neuropathy^22^ and fibromyalgia.^23^ Data on the effect of vortioxetine in models of inflammatory pain are controversial. Vortioxetine did not change pain thresholds in the complete Freund adjuvant (CFA) model of chronic inflammatory pain,^20^ but reduced nocifensive behaviour in the orofacial formalin and acetic acid-writhing tests in mice and mechanical hyperalgesia in the carrageenan model of inflammation in rats.^24^

These studies laid the groundwork for the evaluation of the analgesic activity of vortioxetine in patients suffering from comorbid depression and pain. In two open-label studies, vortioxetine showed analgesic activity in patients suffering from burning mouth syndrome.^25,26^ In a multicentre, prospective, open label study, vortioxetine treatment induced analgesia in patients with major depressive disorder (MDD) associated with chronic pain.^27^ Thus, the use of vortioxetine may expand the therapeutic options in the treatment of MDD and comorbid neuropathic pain. What is missing in preclinical studies is a head-to-head comparison between vortioxetine and other antidepressants in a model of neuropathic pain. The role of TCAs and SNRIs in pain treatment is well acknowledged in both preclinical and clinical studies, whereas vortioxetine has not yet clinical indications for the treatment of neuropathic pain. Moreover, there are no preclinical studies in which the different classes of antidepressants are compared. Here, we examined the effect of a 2-week treatment with vortioxetine, amitriptyline, duloxetine, fluoxetine and paroxetine on mechanical and thermal pain thresholds, and depression- and anxiety-like behaviour in the streptozotocin model of painful diabetic neuropathy, in which vortioxetine was reported to be effective.^22^

## Materials and methods

### Induction of experimental diabetes in mice and drug treatments

We used two-month-old male C57Bl/6 mice (bred in the animal house of I.R.C.C.S. Neuromed) for the induction of diabetic neuropathy. Mice were kept under controlled conditions (*T* = 22°C; humidity = 40%) on a 12-h light-dark cycle with food and water *ad libitum*. Diabetes mellitus was induced by a single injection of streptozotocin (STZ; 200 mg/kg, intraperitoneal (i.p.), as described by Furman).^23^ Blood glucose levels were measured with a glucometer (ACCU-CHEK Active, Roche), according to the manufacturer’s instructions. Mice with random blood glucose levels ≥ 250 mg/dl 13 days after STZ injection were considered as diabetic and used for the assessment of mechanical pain thresholds the following day. Age-matched, non-diabetic C57Bl/6 mice were used as controls. Randomized groups of 7/17 diabetic or non-diabetic mice were treated i.p. as follows: 14 days injections of saline, vortioxetine 10 mg/kg (provided by Lundbeck A/S, Denmark), fluoxetine hydrochloride 10 mg/kg #F132, paroxetine hydrochloride 10 mg/kg #PHR1804, amitriptyline hydrochloride 10 mg/kg #A8404 and duloxetine hydrochloride 10 mg/kg #PHR1865 were purchased by Merck (KGaA Darmstadt, Germany). The fixed dose of 10 mg/kg for the five drugs was selected on the basis of previous studies in models of neuropathic pain.^20,28–30^

### Body weight and glucose levels

Body weight was measured at the beginning (day 0) and at the end of the experiment (14 days after diabetes induction), prior to the evaluation of glucose levels. We evaluated glucose levels at day 0 (before intraperitoneal injection of STZ or vehicle), at day 2 and at day 14 of the experiment, on blood drops obtained by tail tip puncture.

Blood glucose levels were measured with a glucometer according to the manufacturer’s instructions. Mice with random blood glucose levels ≥ 250 mg/dl 14 days after STZ injection were considered as diabetic and used for the assessment of mechanical pain thresholds.

### Behavioural tests

The behavioural tests were carried out in a specific dark testing room, between 2 and 4 PM. All experiments were conducted in a blinded manner, with the researchers unaware of the treatment group assignments.

### Pain testing/assessment of mechanical pain thresholds

Mechanical pain thresholds were quantified by measuring the hind paw withdrawal response to von Frey filament stimulation. In brief, mice were placed in a plexiglass box (20 cm high, 9 cm diameter) with a wire grid bottom through which the von Frey filaments (North Coast Medical, Inc., San Jose, CA; bending force range from 0.008 to 3.5 g) were applied by using a modified version of the up-down paradigm. The filaments were applied five times each, in order of increasing forces, and pressed perpendicularly to the plantar surface of the hind paw until they bent. The first filament that evoked at least three responses was assigned as the pain threshold in grams.

### Pain testing/thermal assay

The hot plate test was used to measure thermal pain threshold. Mice were placed on a 50°C heated surface (Ugo Basile, Gemonio (VA), Italy); the time to the first sign of nociception, such as licking of the back paws or jumping, was recorded and the animal was immediately removed.

### Light-dark box

Thirteen days after the induction of diabetes mice were tested for anxiety using the light-dark box test. The light-dark apparatus consisted of an acrylic box of 20 × 50 × 20 cm, divided into a dark compartment (one third) and an illuminated compartment (two thirds). The division between zones contained a door of 12 × 5 cm, which permitted the passage of the animal from the white to the black compartment. The test was carried out as previously described,^31^ between 9:30 AM and 5:00 PM. Mice were placed inside the dark compartment facing away from the access door and were recorded for 10 min. The number of entries into the light compartment, the time spent in the light compartment, and the number of transitions between the compartments were recorded.

### Tail suspension test

Fourteen days after the induction of diabetes, mice were tested for depressive-like behaviour using the tail suspension test. On testing day, mice were moved to the testing room 1 h prior. Mice were suspended by the tail from a hook connected to a horizontal steel bar (the distance from floor was 20 cm) using adhesive tape, in a constant position three quarters of the distance from the base of the tail. To avoid injury, the animals were suspended by passing the suspension hook through the adhesive tape as close as possible to the tail (1–2 mm) to ensure that the animal hangs with its tail in a straight line. The mice were videotaped manually for 6 min and the parameter recorded was the immobility time (sec) in the last 4 min and the latency (sec) to immobility.^32^

### Western blot analysis

Western blot analysis was performed in the dorsal regions of the lumbar spinal cord dissected from diabetic or non-diabetic mice.

Tissue was homogenized on ice with RIPA buffer containing protease and phosphatase inhibitors cocktail tablet (Santa Cruz Biotechnology, Inc., Temecula, CA). Homogenates were centrifuged at 14,000 r/min for 10 min, and an aliquot was used for protein determinations. Equal amounts of proteins (20 µg) from supernatants were separated by 8/10% SDS polyacrylamide gel and transferred on Immuno PVDF membranes (Bio-Rad, Milan, Italy) for 7 min using Trans Blot Turbo System (Bio-Rad, Milan, Italy). Membranes were blocked overnight in blocking buffer (TBS, 0.05% Tween-20 and 5% non-fat dry milk) at 4°C. Membranes were incubated with the following primary antibody: anti-5HT_3A_ receptors (1:2000, Sigma-Aldrich, Darmstadt, Germany).

Blots were incubated with corresponding secondary antibody. The blots were re-probed with anti-β-Actin monoclonal antibody (1:50,000 Sigma-Aldrich). Immunostaining was revealed by enhanced chemiluminescence luminosity (AmershamPharmacia Biotech, Arlington Height, IL) and images captured by ChemiDoc System (BioRad, Berkley, CA).

### Statistical analysis

Statistical analysis was carried out by Student’s *t*-test for blood glucose levels and protein determinations, One-Way Analysis of Variance (ANOVA) or Two-Way ANOVA for repeated measures followed by Fisher’s LSD multiple comparisons test for nocifensive, anxiety-like and depressive-like behaviours. *p* values < 0.05 were considered significant. The Grubbs test was used for the identification of outlier values.

## Results

**Figure 1.**
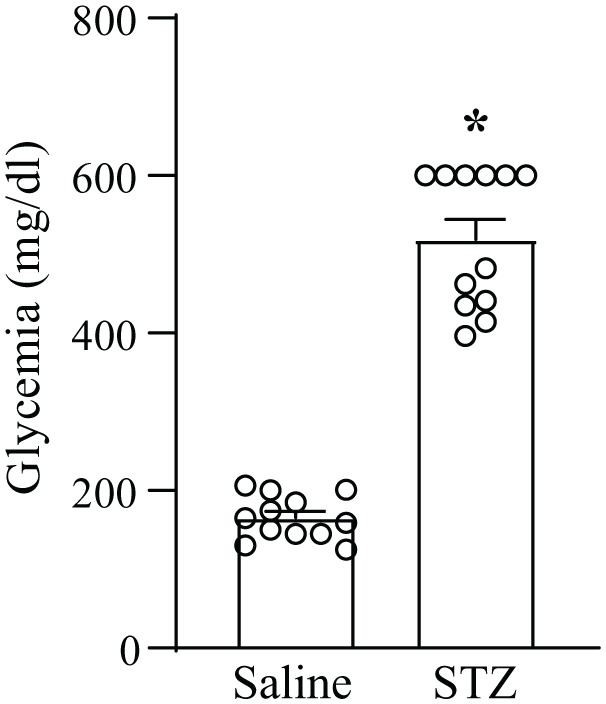
Blood glucose levels in mice injected i.p. with either saline or streptozotocin (STZ, 200 mg/kg). Values are means + S.E.M. of 12 mice per group *p < 0.05 (Student’s *t* test), *t* = 13.41.

For the induction of painful diabetic neuropathy, we injected mice with a single dose of STZ (200 mg/kg, i.p.). Measurements of blood glucose levels confirmed the induction of diabetes 14 days after STZ injection (Figure 1).

**Figure 2.**
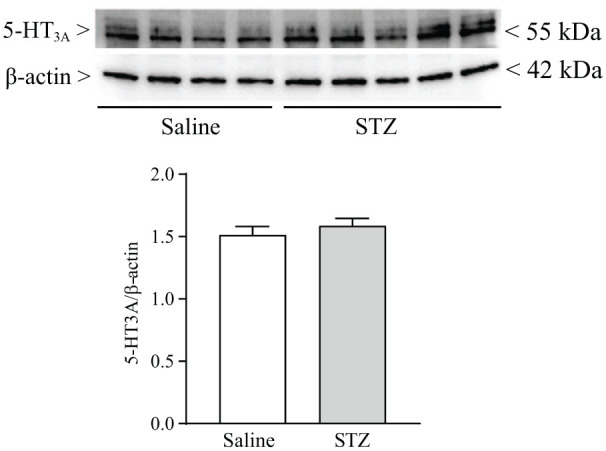
Streptozotocin (STZ)-induced diabetes did not cause changes in 5-HT receptors in the dorsal region of the lumbar spinal cord. Values are means + S.E.M. of four mice in the Saline group and five mice in the STZ group.

In subgroups of mice, we measured protein levels of 5-HT_3A_ receptors in the dorsal portion of the lumbar spinal cord. 5-HT_3A_ receptor protein levels did not change 14 days after STZ injection (Figure 2).

**Figure 3.**
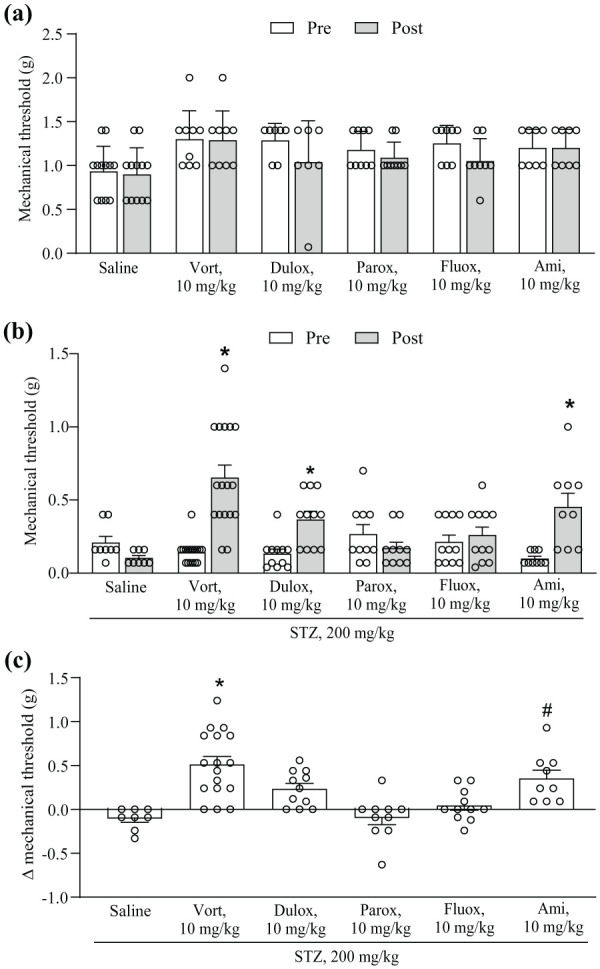
Mechanical pain thresholds in control (a) or streptozotocin (STZ) (b) mice treated for 2 weeks with saline, vortioxetine (Vort), duloxetine (Dulox), paroxetine (Parox), fluoxetine (Fluox) or amitriptyline (Ami). Thresholds were measured 24 h before (Pre) and at the end (Post) of drug treatment. In (a), values are means + S.E.M. of 12 saline, 9 Vort, 7 Dulox, 9 Parox, 8 Fluox and 8 Ami mice per group. In (b) values are means + S.E.M. of 8 saline, 17 Vort, 11 Dulox, 10 Parox, 11 Fluox and 9 Ami mice per group. * *p* < 0.05 versus the respective ‘Pre’ values (Two-Way ANOVA for repeated measures + Fisher’s LSD multiple comparisons test); time factor (Post vs Pre): F_(1,60)_ = 21.71; *p* < 0.0001; treatment factor: F_(5,60)_ = 4.544; *p* = 0.0014; interaction: F_(5,60)_ = 10.56; *p* < 0.0001. (c) Δ mechanical pain thresholds in STZ mice. *p < 0.05 versus saline, parox and fluox; #*p* < 0.05 versus saline and parox (One-Way ANOVA + Fisher’s LSD multiple comparisons test), F_(5,60)_ = 10.56; *p* < 0.0001.

Control and diabetic mice were treated daily i.p. for 2 weeks with either saline, vortioxetine, duloxetine, fluoxetine, paroxetine or amitriptyline (all drugs at the dose of 10 mg/kg), starting 2 weeks following saline (in controls) or STZ injection. In control non-diabetic mice none of the antidepressants caused significant changes in mechanical pain thresholds after 2 weeks of treatment (Figure 3(a)). STZ injection largely reduced mechanical pain thresholds after 2 weeks, before treatment with either saline or antidepressant drugs. Means + S.E.M. of all ‘Pre’ values in control and STZ-injected mice (Figure 3(a) and 3(b)) are 1.17 + 0.04 and 0.17 + 0.016, respectively (*n* = 53 and 66 in controls and STZ-injected mice; *p* < 0.0001; Student’s *t* test). Treatments with vortioxetine, duloxetine or amitriptyline significantly enhanced pain thresholds, whereas treatments with saline, fluoxetine or paroxetine did not reduce mechanical pain (Figure 3(b)). Vortioxetine reduced mechanical pain to a greater extent than duloxetine and amitriptyline (Figure 3(c)).

**Figure 4.**
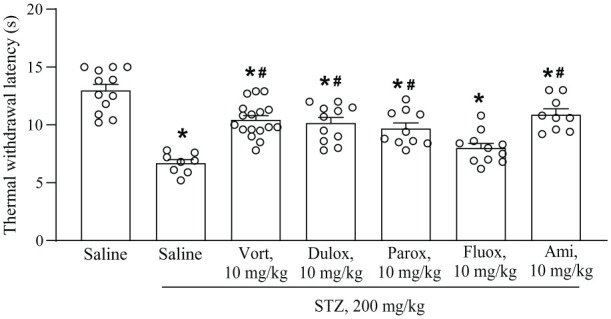
Thermal pain thresholds in non-diabetic mice treated for 2 weeks with saline or streptozotocin (STZ) mice treated for 2 weeks with saline, vortioxetine (Vort), duloxetine (Dulox), paroxetine (Parox), fluoxetine (Fluox) or amitriptyline (Ami). Values are means + S.E.M. of 12 saline, 8 saline STZ, 17 Vort, 11 Dulox, 10 Parox, 11 Fluox and 9 Ami mice per group. *p* < 0.05 versus values obtained in non-diabetic mice treated with saline (*) or versus diabetic mice treated with saline or fluoxetine (#) (One-Way ANOVA + Fisher’s LSD multiple comparisons test), F_(6,71)_ = 18.47; *p* < 0.001.

Thermal latencies were measured in non-diabetic mice treated with saline and in all groups of diabetic mice. Thermal latencies were significantly reduced in all groups of STZ-injected mice with respect to control mice treated with saline (Figure 4). In STZ-injected mice, treatments with vortioxetine, duloxetine or amitriptyline significantly increased thermal latencies with respect to mice treated with either saline or fluoxetine, which was the only antidepressant lacking an effect on thermal latencies. Treatment with paroxetine increased thermal thresholds with respect to treatment with saline, but not with fluoxetine (Figure 4).

**Figure 5.**
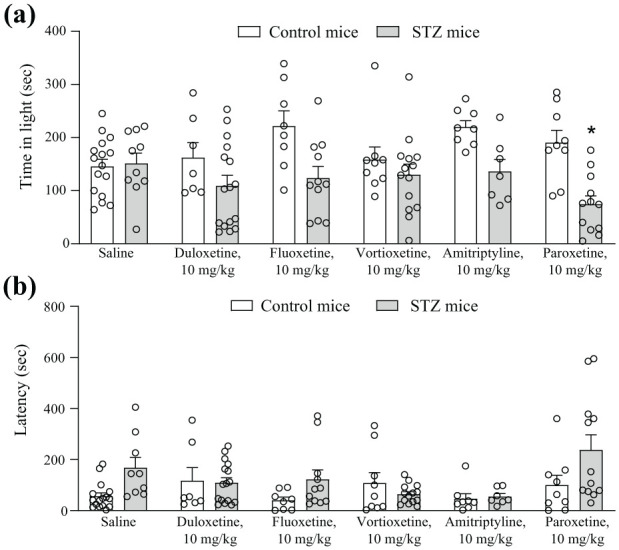
Anxiety-like behaviour in control and streptozotocin (STZ) mice. The time spent in the light compartment of the light-dark box is shown in (a). Latencies to enter the light compartment for the first time are shown in (b). Values are mean + S.E.M. of 16 saline, 9 Vortioxetine, 7 Duloxetine, 9 Paroxetine, 8 Fluoxetine and 8 Amitriptyline mice per group for control mice and 10 saline, 14 Vortioxetine, 16 Duloxetine, 12 Paroxetine, 11 Fluoxetine and 7 Amitriptyline mice per group for STZ mice. In the evaluation of latency one value of the STZ groups treated with saline was identified as outlier by the Grubbs test and was removed from the analysis. *p < 0.05 versus the respective control (Two-Way ANOVA + Fisher’s LSD test). In (a), control versus STZ: F_(1,115)_ = 25.22; *p* < 0.0001; treatment factor: F_(5,115)_ = 1.435; *p* = 0.2168; interaction: F_(5,115)_ = 2.522; *p* = 0.0332. In (b), control versus STZ: F_(1,114)_ = 5.967; *p* = 0.163; treatment: F(_5,114)_ = 2.655; *p* = 0.0262; interaction: F_(5,114)_ = 2.441; *p* = 0.0384.

We assessed risk-taking (or anxiety-like) behaviour in control or diabetic mice using the light/dark box test, in which mice are considered more ‘anxious’ if they avoid the light compartment. In saline treated mice, STZ injection did not significantly change the total time spent in the light compartment and the latency to enter the light compartment of the light-dark box (Figure 5(a) and (b)). None of the treatments caused behavioural changes in the light-dark box test with the exception of paroxetine, which, unexpectedly, reduced risk-taking behaviour (i.e. increased anxiety-like behaviour; Figure 5(a) and (b)).

**Figure 6.**
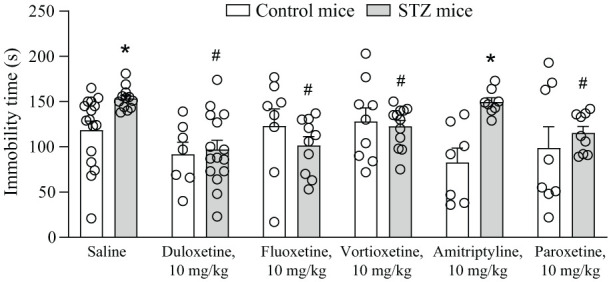
Depressive-like behaviour in control and streptozotocin (STZ) mice. Values are mean + S.E.M. of 16 saline, 9 Vortioxetine, 7 Duloxetine, 8 Paroxetine, 8 Fluoxetine and 8 Amitriptyline mice per group for control mice and 12 saline, 12 Vortioxetine, 15 Duloxetine, 9 Paroxetine, 10 Fluoxetine and 8 Amitriptyline mice per group for STZ mice. *p* < 0.05 versus the respective control (*) or versus STZ/saline (#) (Two-Way ANOVA + Fisher’s LSD test). Control versus STZ: F_(1,109)_ = 5.255; *p* = 0.238; treatment factor: F_(5,109)_ = 3.316; *p* = 0.0079; interaction: F_(5,109)_ = 3.045; *p* = 0.0130.

Finally, we measured the immobility time in the tail suspension test to assess depression-like behaviour. STZ injection in saline-treated mice significantly enhanced the immobility time with respect to control mice treated with saline (i.e. STZ induced depressive-like behaviour). This effect was abolished by all antidepressants except amitriptyline (Figure 6).

## Discussion

Diabetes mellitus (DM) is an endocrine disorder characterized by either the lack of insulin (in type-1 DM) or the development of insulin resistance (in type-2 DM). This leads to increases in blood glucose levels, abnormalities in fat and protein metabolism and a series of long-term complications, which severely compromise the quality of life of DM patients.

Diabetic polyneuropathy, one of the most common complications of both type-1 and -2 DM, is characterized by a length-dependent damage of peripheral nerves, and alterations of limb sensations and pain, and often persists in spite of a good control of blood glucose levels.^33,34^ Depression is frequently associated with diabetic neuropathy, and the severity of depressive symptoms is directly related to pain intensity.^35–37^

For this reason, antidepressant drugs that are known to reduce neuropathic pain, such as amitriptyline and duloxetine, are first-line drugs in the treatment of diabetic neuropathy,^2^ particularly in patients with comorbid depression. In contrast, SSRIs, such as fluoxetine and paroxetine, show level A/B rating for inefficacy or discrepant results in diabetic neuropathy.^2^ However, SNRIs, including duloxetine, and amitriptyline may increase the risk for hypertriglyceridemia and metabolic syndrome,^38^ which play a key role in the pathophysiology of diabetic neuropathy, and this limits the therapeutic use of these antidepressants. Vortioxetine might be considered a valuable option in the treatment of diabetic neuropathy and comorbid depression because this drug has consistently shown analgesic activity in animal models of neuropathic pain, including the STZ model of diabetes (see Introduction and References therein). Vortioxetine is widely used in the treatment of major depressive disorder with a remarkable effect on cognitive symptoms, anhedonia, and emotional blunting, and excellent profile of safety and tolerability and lack of pharmacokinetic drug-drug interactions.^19,39^

Here, we compared vortioxetine with duloxetine, amitriptyline and the two SSRIs, fluoxetine and paroxetine, on pain thresholds and depressive-like and anxiety-like behaviour in the STZ model of painful diabetic neuropathy. We confirmed previous findings^22^ showing that vortioxetine treatment caused analgesia in diabetic mice measuring both mechanical and thermal pain thresholds. Interestingly, vortioxetine enhanced mechanical thresholds in diabetic mice to a greater extent than duloxetine and amitriptyline (see Figure 3(c)), although the three drugs equally enhanced thermal pain thresholds. This suggests that the potent 5-HT_3_ receptor blockade by vortioxetine has a stronger impact on mechanical thresholds, or, alternatively, that the three drugs caused a ceiling effect on thermal pain. Neither fluoxetine nor paroxetine had any effect on mechanical pain, suggesting that drugs that enhance synaptic serotonin levels without interacting with serotonin receptors (with the exception of 5-HT_2C_ receptors for fluoxetine) have no effect on chronic pain. This is consistent with the evidence that chronic pain is associated with functional changes in serotonergic neurons projecting to the spinal cord, which contribute to nociceptive sensitization reducing pain thresholds.^7,8^ Unexpectedly, however, paroxetine enhanced thermal pain thresholds in diabetic mice, albeit to a lesser extent than vortioxetine, duloxetine or amitriptyline. We have no explanation for the differential effect of fluoxetine and paroxetine on thermal pain in diabetic mice because both drugs display a great potency as inhibitors of serotonin transporter. Paroxetine also acts as a muscarinic cholinergic receptor antagonist at high doses, but it is unlikely that this mechanism might have contributed to the effect of paroxetine on thermal pain because it is the activation of muscarinic receptors that causes analgesia.^40–42^

Data obtained with the light-dark box and tail suspension test showed that diabetic mice displayed depressive-like behaviour but no changes in risk-taking (or anxiety-like) behaviour. We were surprised to find that chronic treatment with paroxetine reduced risk-taking behaviour in STZ mice because paroxetine is indicated for the treatment of anxiety in humans.^43^ Treatments with all antidepressants reversed the increase in depressive-like behaviour in diabetic mice, with the exception of amitriptyline. This was also surprising in light of the robust antidepressant effect displayed by amitriptyline in rodents.^44^ It is possible that neuroadaptive mechanisms associated with diabetes alter behavioural responses to classical anxiolytic and antidepressant drugs, such as paroxetine and amitriptyline. For example, it has been shown that a single injection of fluoxetine is less effective in reducing the immobility time in the forced swim test in diabetic mice because the component mediated by 5-HT_1A_ receptors in the antidepressant activity of acute fluoxetine treatment is lost in diabetic mice.^45^ The identification of the neuroadaptive mechanisms underlying the paradoxical anxiolytic effect of paroxetine and the lack of antidepressant-like effect of amitriptyline in diabetic mice awaits further investigation.

In conclusion, our findings support the analgesic activity of vortioxetine in the STZ model of painful diabetic neuropathy, an effect that may involve more than one mechanism. 5-HT_3_ receptor levels in the spinal were unchanged in diabetic mice, and vortioxetine-induced analgesia might be consequent to 5-HT_3_ receptor blockade. However, vortioxetine also behaves as a full agonist of 5-HT_1A_ receptor, a partial agonist of 5-HT_1B_ receptors, and antagonist of 5-HT_1D_ and 5-HT_7_ receptor and a SERT inhibitor (see Introduction and References therein). All these targets might contribute to the overall analgesic action of vortioxetine. It will be important to examine whether and in which direction induction of diabetes with STZ influences the expression of the vortioxetine targets in different stations of the pain neuraxis.

Vortioxetine may be a valuable drug in the treatment of diabetic neuropathy and comorbid depression owing to its combined antidepressant and analgesic activity. Diabetes is associated with both cognitive dysfunction and depression as a result of cerebral microvascular complications.^46^ This may reinforce the choice of vortioxetine in the treatment of diabetes and comorbid depression patients because the drug shows superiority with respect to all other antidepressants in improving cognitive functions.^19,47^ In addition, vortioxetine displays an excellent profile of cardiovascular safety, and causes no changes in body weight or sexual dysfunction.^19^ As opposed to fluoxetine, paroxetine and duloxetine, vortioxetine is not an inhibitor of CYP2D6 or other isoforms of cytochrome-P_450_,^48^ and, therefore, lacks pharmacokinetic interactions with other drugs used in the treatment of diabetic neuropathy. It is the right time to investigate the efficacy of vortioxetine in patients affected by diabetic neuropathy.

## References

[1] Moulin DE Clark AJ Gilron I Ware MA Watson CP Sessle BJ Coderre T Morley-Forster PK Stinson J Boulanger A Peng P Finley GA Taenzer P Squire P Dion D Cholkan A Gilani A Gordon A Henry J Jovey R Lynch M Mailis-Gagnon A Panju A Rollman GB Velly A ; Canadian Pain Society. Pharmacological management of chronic neuropathic pain - consensus statement and guidelines from the Canadian Pain Society. Pain Res Manag 2007; 12(1): 13–21. 17372630 10.1155/2007/730785 PMC2670721

[2] Attal N Cruccu G Baron R Haanpää M Hansson P Jensen TS Nurmikko T . EFNS guidelines on the pharmacological treatment of neuropathic pain: 2010 revision. Eur J Neurol 2010; 17(9): 1113-e88. 10.1111/j.1468-1331.2010.02999.x 20402746

[3] Attal N . Pharmacological treatments of neuropathic pain: the latest recommendations. Rev Neurol (Paris) 2019; 175(1–2): 46–50. 30318260 10.1016/j.neurol.2018.08.005

[4] Bates D Schultheis BC Hanes MC Jolly SM Chakravarthy KV Deer TR Levy RM Hunter CW . A comprehensive algorithm for management of neuropathic pain. Pain Med 2019; 20(Suppl 1): S2–S12. Erratum in: *Pain Med* 2023; 24(2): 219. 10.1093/pm/pnz075 PMC6544553 31152178

[5] Leo RJ Barkin RL . Antidepressant use in chronic pain management: is there evidence of a role for duloxetine? Prim Care Companion J Clin Psychiatry 2003; 5(3): 118–123. 15154022 10.4088/pcc.v05n0303 PMC406378

[6] Matejowsky HG Kataria S Spillers NJ O’Quin CC Barrie S Ahmadzadeh S Shekoohi S Kaye AD . Interventional procedures for refractory neuropathic pain. Explor Neurosci 2023; 2: 276–286.

[7] Wei F Dubner R Zou S Ren K Bai G Wei D Guo W . Molecular depletion of descending serotonin unmasks its novel facilitatory role in the development of persistent pain. J Neurosci 2010; 30(25): 8624–8636. 20573908 10.1523/JNEUROSCI.5389-09.2010 PMC2902253

[8] Zhang Y Li A Lao L Xin J Ren K Berman BM Zhang RX . Rostral ventromedial medulla μ, but not κ, opioid receptors are involved in electroacupuncture anti-hyperalgesia in an inflammatory pain rat model. Brain Res 2011; 1395: 38–45. 21565329 10.1016/j.brainres.2011.04.037 PMC3105222

[9] Oatway MA Chen Y Weaver LC . The 5-HT3 receptor facilitates at-level mechanical allodynia following spinal cord injury. Pain 2004; 110(1–2): 259–268. 15275776 10.1016/j.pain.2004.03.040

[10] Chen Y Oatway MA Weaver LC . Blockade of the 5-HT3 receptor for days causes sustained relief from mechanical allodynia following spinal cord injury. J Neurosci Res 2009; 87(2): 418–424. 18798253 10.1002/jnr.21860

[11] Patel R Dickenson AH . Modality selective roles of pro-nociceptive spinal 5-HT2A and 5-HT3 receptors in normal and neuropathic states. Neuropharmacology 2018; 143: 29–37. 30240783 10.1016/j.neuropharm.2018.09.028 PMC6277848

[12] Neziri AY Dickenmann M Scaramozzino P Andersen OK Arendt-Nielsen L Dickenson AH Curatolo M . Effect of intravenous tropisetron on modulation of pain and central hypersensitivity in chronic low back pain patients. Pain 2012; 153(2): 311–318. 22100357 10.1016/j.pain.2011.10.008

[13] Li ZL Xue Y Tao ZY Du WZ Jiang YG Cao DY . Spinal 5-HT3 receptor contributes to somatic hyperalgesia induced by sub-chronic stress. Mol Pain 2019; 15: 1744806919859723. 10.1177/1744806919859723 PMC6613060 31184246

[14] Heijmans L Mons MR Joosten EA . A systematic review on descending serotonergic projections and modulation of spinal nociception in chronic neuropathic pain and after spinal cord stimulation. Mol Pain 2021; 17: 17448069211043965. 10.1177/17448069211043965 PMC8527581 34662215

[15] McCleane GJ Suzuki R Dickenson AH . Does a single intravenous injection of the 5HT3 receptor antagonist ondansetron have an analgesic effect in neuropathic pain? A double-blinded, placebo-controlled cross-over study. Anesth Analg 2003; 97(5): 1474–1478. 14570668 10.1213/01.ANE.0000085640.69855.51

[16] Ladefoged LK Munro L Pedersen AJ Lummis SCR Bang-Andersen B Balle T Schiøtt B Kristensen AS . Modeling and mutational analysis of the binding mode for the multimodal antidepressant drug vortioxetine to the human 5-HT3A receptor. Mol Pharmacol 2018; 94(6): 1421–1434. 30257860 10.1124/mol.118.113530

[17] Stahl SM . Modes and nodes explain the mechanism of action of vortioxetine, a multimodal agent (MMA): enhancing serotonin release by combining serotonin (5HT) transporter inhibition with actions at 5HT receptors (5HT1A, 5HT1B, 5HT1D, 5HT7 receptors). CNS Spectr 2015; 20(2): 93–97. 25831967 10.1017/S1092852915000139

[18] Gonda X Sharma SR Tarazi FI . Vortioxetine: a novel antidepressant for the treatment of major depressive disorder. Expert Opin Drug Discov 2019; 14(1): 81–89. 30457395 10.1080/17460441.2019.1546691

[19] Lam RW Kennedy SH Adams C Bahji A Beaulieu S Bhat V Blier P Blumberger DM Brietzke E Chakrabarty T Do A Frey BN Giacobbe P Gratzer D Grigoriadis S Habert J Ishrat Husain M Ismail Z McGirr A McIntyre RS Michalak EE Müller DJ Parikh SV Quilty LS Ravindran AV Ravindran N Renaud J Rosenblat JD Samaan Z Saraf G Schade K Schaffer A Sinyor M Soares CN Swainson J Taylor VH Tourjman SV Uher R van Ameringen M Vazquez G Vigod S Voineskos D Yatham LN Milev RV . Canadian Network for Mood and Anxiety Treatments (CANMAT) 2023 update on clinical guidelines for management of major depressive disorder in adults: Réseau canadien pour les traitements de l’humeur et de l’anxiété (CANMAT) 2023: Mise à jour des lignes directrices cliniques pour la prise en charge du trouble dépressif majeur chez les adultes. Can J Psychiatry 2024; 69(9): 641–687. 38711351 10.1177/07067437241245384 PMC11351064

[20] Zuena AR Maftei D Alemà GS Dal Moro F Lattanzi R Casolini P Nicoletti F . Multimodal antidepressant vortioxetine causes analgesia in a mouse model of chronic neuropathic pain. Mol Pain 2018; 14: 1744806918808987. 10.1177/1744806918808987 PMC6207957 30289053

[21] Micov AM Tomić MA Todorović MB Vuković MJ Pecikoza UB Jasnic NI Djordjevic JD Stepanović-Petrović RM . Vortioxetine reduces pain hypersensitivity and associated depression-like behavior in mice with oxaliplatin-induced neuropathy. Prog Neuropsychopharmacol Biol Psychiatry 2020; 103: 109975. 32464241 10.1016/j.pnpbp.2020.109975

[22] Turan Yücel N Kandemir Ü Üçel Uİ Demir Özkay Ü Can ÖD . Catecholaminergic and cholinergic systems mediate beneficial effect of vortioxetine on diabetes-induced neuropathic pain. Biomedicines 2023; 11(4): 1137. 37189755 10.3390/biomedicines11041137 PMC10135813

[23] Furman BL . Streptozotocin-induced diabetic models in mice and rats. Curr Protoc 2021; 1(4): e78. 10.1002/cpz1.78 33905609

[24] Sałat K Furgała-Wojas A . Serotonergic neurotransmission system modulator, vortioxetine, and dopaminergic D2/D3 receptor agonist, ropinirole, attenuate fibromyalgia-like symptoms in mice. Molecules 2021; 26(8): 2398. 33924258 10.3390/molecules26082398 PMC8074757

[25] Todorović M Micov A Nastić K Tomić M Pecikoza U Vuković M Stepanović-Petrović R . Vortioxetine as an analgesic in preclinical inflammatory pain models: mechanism of action. Fundam Clin Pharmacol 2022; 36(2): 237–249. 34820899 10.1111/fcp.12737

[26] Adamo D Pecoraro G Aria M Favia G Mignogna MD . Vortioxetine in the treatment of mood disorders associated with burning mouth syndrome: results of an open-label, flexible-dose pilot study. Pain Med 2020; 21(1): 185–194. 31343684 10.1093/pm/pnz120

[27] Adamo D Pecoraro G Coppola N Calabria E Aria M Mignogna M . Vortioxetine versus other antidepressants in the treatment of burning mouth syndrome: an open-label randomized trial. Oral Dis 2021; 27(4): 1022–1041. 32790904 10.1111/odi.13602

[28] Jesse CR Wilhelm EA Nogueira CW . Depression-like behavior and mechanical allodynia are reduced by bis selenide treatment in mice with chronic constriction injury: a comparison with fluoxetine, amitriptyline, and bupropion. Psychopharmacology (Berl) 2010; 212: 513–522. 20689938 10.1007/s00213-010-1977-6

[29] Zychowska M Rojewska E Makuch W Przewlocka B Mika J . The influence of microglia activation on the efficacy of amitriptyline, doxepin, milnacipran, venlafaxine and fluoxetine in a rat model of neuropathic pain. Eur J Pharmacol 2015; 749: 115–123. 25460025 10.1016/j.ejphar.2014.11.022

[30] Yoneda S Kasai E Matsuo M Tamano R Sakurai Y Asaki T Fujita M . Duloxetine ameliorates the impairment of diffuse noxious inhibitory control in rat models of peripheral neuropathic pain and knee osteoarthritis pain. Neurosci Lett 2020; 729: 134990. 32315711 10.1016/j.neulet.2020.134990

[31] Finger BC Dinan TG Cryan JF . Leptin-deficient mice retain normal appetitive spatial learning yet exhibit marked increases in anxiety-related behaviours. Psychopharmacology (Berl) 2010; 210(4): 559–568. 20422404 10.1007/s00213-010-1858-z

[32] Steru L Chermat R Thierry B Simon P . The tail suspension test: a new method for screening antidepressants in mice. Psychopharmacology (Berl) 1985; 85(3): 367–370. 3923523 10.1007/BF00428203

[33] Feldman EL Callaghan BC Pop-Busui R Zochodne DW Wright DE Bennett DL Bril V Russell JW Viswanathan V . Diabetic neuropathy. Nat Rev Dis Primers 2019; 5(1): 42. 31197183 10.1038/s41572-019-0097-9 PMC7096070

[34] Eid SA Rumora AE Beirowski B Bennett DL Hur J Savelieff MG Feldman EL . New perspectives in diabetic neuropathy. Neuron 2023; 111(17): 2623–2641. 37263266 10.1016/j.neuron.2023.05.003 PMC10525009

[35] Gore M Brandenburg NA Dukes E Hoffman DL Tai KS Stacey B . Pain severity in diabetic peripheral neuropathy is associated with patient functioning, symptom levels of anxiety and depression, and sleep. J Pain Symptom Manage 2005; 30(4): 374–385. 16256902 10.1016/j.jpainsymman.2005.04.009

[36] Gore M Brandenburg NA Hoffman DL Tai KS Stacey B . Burden of illness in painful diabetic peripheral neuropathy: the patients’ perspectives. J Pain 2006; 7(12): 892–900. 17157775 10.1016/j.jpain.2006.04.013

[37] Jain R Jain S Raison CL Maletic V . Painful diabetic neuropathy is more than pain alone: examining the role of anxiety and depression as mediators and complicators. Curr Diab Rep 2011; 11(4): 275–284. 21611765 10.1007/s11892-011-0202-2

[38] Gramaglia C Gambaro E Bartolomei G Camera P Chiarelli-Serra M Lorenzini L Zeppegno P . Increased risk of metabolic syndrome in antidepressants users: a mini review. Front Psychiatry 2018; 9: 621. 30546325 10.3389/fpsyt.2018.00621 PMC6279880

[39] Cuomo A Aguglia A De Berardis D Ventriglio A Gesi C Fagiolini A . Individualized strategies for depression: narrative review of clinical profiles responsive to vortioxetine. Ann Gen Psychiatry 2024; 23(1): 20. 38755657 10.1186/s12991-024-00505-1 PMC11097484

[40] Shannon HE Womer DE Bymaster FP Calligaro DO DeLapp NC Mitch CH Ward JS Whitesitt CA Swedberg MD Sheardown MJ Fink-Jensen A Olesen PH Rimvall K Sauerberg P . In vivo pharmacology of butylthio[2.2.2] (LY297802 / NNC11-1053), an orally acting antinociceptive muscarinic agonist. Life Sci 1997; 60(13–14): 969–976. 9121363 10.1016/s0024-3205(97)00036-2

[41] Wess J Duttaroy A Gomeza J Zhang W Yamada M Felder CC Bernardini N Reeh PW . Muscarinic receptor subtypes mediating central and peripheral antinociception studied with muscarinic receptor knockout mice: a review. Life Sci 2003; 72(18–19): 2047–2054. 12628455 10.1016/s0024-3205(03)00082-1

[42] Tata AM . Muscarinic acetylcholine receptors: new potential therapeutic targets in antinociception and in cancer therapy. Recent Pat CNS Drug Discov 2008; 3(2): 94–103. 18537768 10.2174/157488908784534621

[43] Slee A Nazareth I Bondaronek P Liu Y Cheng Z Freemantle N . Pharmacological treatments for generalised anxiety disorder: a systematic review and network meta-analysis. Lancet 2019; 393(10173): 768–777. 30712879 10.1016/S0140-6736(18)31793-8

[44] Pandey DK Mahesh R Kumar AA Rao VS Arjun M Rajkumar R . A novel 5-HT(2A) receptor antagonist exhibits antidepressant-like effects in a battery of rodent behavioural assays: approaching early-onset antidepressants. Pharmacol Biochem Behav 2010; 94(3): 363–373. 19800913 10.1016/j.pbb.2009.09.018

[45] Miyata S Hirano S Kamei J . Diabetes attenuates the antidepressant-like effect mediated by the activation of 5-HT1A receptor in the mouse tail suspension test. Neuropsychopharmacology 2004; 29(3): 461–469. 14628002 10.1038/sj.npp.1300354

[46] van Sloten TT Sedaghat S Carnethon MR Launer LJ Stehouwer CDA . Cerebral microvascular complications of type 2 diabetes: stroke, cognitive dysfunction, and depression. Lancet Diabetes Endocrinol 2020; 8(4): 325–336. 32135131 10.1016/S2213-8587(19)30405-X PMC11044807

[47] Baune BT Brignone M Larsen KG . A network meta-analysis comparing effects of various antidepressant classes on the Digit Symbol Substitution Test (DSST) as a measure of cognitive dysfunction in patients with major depressive disorder. Int J Neuropsychopharmacol 2018; 21(2): 97–107. 29053849 10.1093/ijnp/pyx070 PMC5793828

[48] Spina E Santoro V . Drug interactions with vortioxetine, a new multimodal antidepressant. Riv Psichiatr 2015; 50(5): 210–215. 26489070 10.1708/2040.22160

